# Breast cancer adaptation to oxygen stress: mechanisms of tumor survival, progression, and therapeutic resistance

**DOI:** 10.3389/or.2026.1959034

**Published:** 2026-10-16

**Authors:** Emmanuel Ifeanyi Obeagu, Izuchukwu Hilary Edeoga

**Affiliations:** 1 Department of Biomedical and Laboratory Science, Africa University, Mutare, Zimbabwe; 2 Department of Haematology and Molecular Medicine, Faculty of Health Sciences, University of the Witwatersrand, Johannesburg, South Africa; 3 Department of Hematology and Immohematology, Bahir Dar University, Bahir Dar, Ethiopia; 4 Department of Medicine and Surgery, Babcock University, Ilishan Remo, Ogun State, Nigeria

**Keywords:** angiogenesis, breast cancer, cell survival, hypoxia, metabolic reprogramming

## Abstract

Breast cancer cells encounter heterogeneous oxygen conditions arising from abnormal vasculature, impaired perfusion, rapid proliferation, and fluctuating blood flow. Adaptation to oxygen stress is increasingly recognized as a major driver of tumor progression and therapeutic resistance. This review integrates current evidence on the mechanisms through which breast cancer cells adapt to oxygen stress and identifies opportunities for precision therapeutic intervention. Oxygen stress activates hypoxia-inducible factors and interconnected signaling networks that coordinate metabolic reprogramming, mitochondrial adaptation, reactive oxygen species generation, and redox homeostasis. These responses are reinforced by autophagy, unfolded protein response, epithelial–mesenchymal plasticity, cancer stemness, extracellular-matrix remodeling, angiogenesis, and immune suppression. Together, these mechanisms sustain cellular survival, facilitate invasion and metastasis, and promote resistance to chemotherapy, radiotherapy, endocrine therapy, HER2-directed treatment, and other targeted approaches. Oxygen-stress responses vary across breast cancer subtypes, emphasizing the need for biologically informed therapeutic strategies. Potential precision approaches include targeting hypoxia signaling, glycolytic and mitochondrial dependencies, lactate metabolism, antioxidant systems, autophagy, proteostasis, hypoxia-activated pathways, and hypoxia-associated immune suppression. Integrating molecular biomarkers, metabolic profiling, spatial technologies, and functional imaging may improve identification of tumors with actionable oxygen-stress dependencies. Oxygen-stress adaptation represents an interconnected survival network rather than a single hypoxia pathway. Therapeutically exploiting tumor-specific adaptive dependencies, while accounting for subtype and spatial heterogeneity, may provide new opportunities to overcome treatment resistance and improve precision management of breast cancer.

## Introduction

Breast cancer remains the most prevalent malignancy among women globally and continues to be a leading cause of cancer-related mortality. Its development and progression are governed not only by genetic mutations and signaling alterations but also by complex interactions with the tumor microenvironment. Among the various microenvironmental stressors, oxygen deprivation—also known as hypoxia—plays a pivotal role in shaping tumor biology. As a solid tumor expands, the existing vasculature often becomes insufficient to meet the oxygen and nutrient demands of proliferating cells, leading to the formation of hypoxic regions within the tumor mass (1–3). Hypoxia is more than just a passive consequence of tumor growth; it is a dynamic factor that actively contributes to cancer aggressiveness, therapeutic resistance, and metastatic potential. The presence of hypoxic zones within breast tumors has been correlated with poor prognosis, increased risk of recurrence, and resistance to conventional therapies such as radiotherapy and chemotherapy. This is due to the ability of cancer cells to adapt to and exploit hypoxic stress through a variety of molecular and cellular mechanisms that enhance their survival and proliferation (4–6). A key regulator of the hypoxic response is the family of hypoxia-inducible factors (HIFs), particularly HIF-1α, which acts as a transcriptional activator for numerous genes involved in angiogenesis, metabolism, pH regulation, and apoptosis. Upon stabilization in low oxygen conditions, HIF-1α translocates to the nucleus and dimerizes with HIF-1β to activate target gene expression. This transcriptional program equips breast cancer cells with tools to cope with the hostile microenvironment and to adapt their physiology to ensure survival (7, 8).

One of the most well characterized hypoxia-induced responses in breast cancer cells is metabolic reprogramming. Normally, cells rely on oxidative phosphorylation for efficient ATP production. However, under hypoxia, cancer cells shift toward glycolysis, a less efficient but oxygen-independent pathway, to sustain their energy needs. This shift, known as the Warburg effect, is further amplified in hypoxic environments, leading to increased glucose uptake and lactate production. This metabolic flexibility allows cancer cells to maintain viability and function even in nutrient- and oxygen-poor regions of the tumor (9, 10). In addition to altering their metabolism, breast cancer cells initiate angiogenesis in response to hypoxia, promoting the formation of new blood vessels to improve oxygen and nutrient delivery. HIF-1α-driven upregulation of vascular endothelial growth factor (VEGF) is central to this process. However, the resulting vasculature is often structurally and functionally abnormal, leading to inefficient perfusion and fluctuating oxygen levels, thereby perpetuating a cycle of hypoxia and further tumor adaptation (11, 12). Beyond metabolic and vascular changes, hypoxia also influences cell survival pathways. Under normal conditions, oxygen stress would trigger apoptosis; however, breast cancer cells can subvert this process. Through the upregulation of anti-apoptotic proteins such as BCL-2 and survivin, and the activation of autophagy pathways, these cells are able to evade cell death. Such adaptations not only facilitate tumor growth but also contribute to resistance against therapy-induced apoptosis, complicating treatment efforts (13, 14).

### Aim

The aim of this narrative review is to explore and synthesize current knowledge on the adaptive responses of breast cancer cells to oxygen stress, with a particular focus on the mechanisms by which these cells survive and proliferate in hypoxic tumor microenvironments.

## Methods (narrative review approach)

This article is based on a narrative review methodology designed to synthesize and critically interpret existing evidence on breast cancer cell adaptation to oxygen stress, with particular emphasis on hypoxia-driven survival mechanisms, tumor progression, and therapeutic resistance.

### Literature search strategy

A comprehensive literature search was conducted across major biomedical databases, including PubMed, Scopus, Web of Science, and Google Scholar. The search covered publications from database inception to 2026. Additional relevant sources were identified through manual screening of reference lists from key review articles and landmark experimental studies. The search strategy used combinations of keywords and Medical Subject Headings (MeSH) terms, including: *breast cancer*, *hypoxia*, *oxygen stress*, *HIF-1α*, *HIF-2α*, *tumor microenvironment*, *metabolic reprogramming*, *angiogenesis*, *oxidative stress*, *epithelial–mesenchymal transition*, and *therapy resistance*.

### Eligibility criteria

Peer-reviewed original research articles, systematic reviews, narrative reviews, experimental studies (*in vitro* and *in vivo*), and clinically relevant translational studies published in English were included. Priority was given to studies published within the last 15–20 years to ensure contemporary relevance, while older foundational studies were included when necessary to support mechanistic concepts. Articles not directly related to hypoxia biology in breast cancer or lacking relevance to oxygen stress–driven mechanisms were excluded.

### Study selection and data handling

Titles and abstracts were screened for relevance to oxygen stress and breast cancer biology. Full-text articles were reviewed to extract key findings related to hypoxia signaling pathways, metabolic adaptation, tumor progression, immune modulation, and treatment resistance. Studies were grouped thematically based on biological processes rather than study design alone.

### Data synthesis

Given the heterogeneity of study designs and outcomes, a qualitative narrative synthesis approach was employed. Evidence was integrated across molecular, cellular, and clinical levels to construct a comprehensive framework describing oxygen stress adaptation in breast cancer.

### Methodological considerations

No quantitative meta-analysis was performed due to variability in experimental models, endpoints, and clinical contexts. Emphasis was placed on conceptual integration and mechanistic interpretation to provide a coherent understanding of hypoxia-driven tumor biology.

### Ethical considerations

This study utilized publicly available literature and did not involve human or animal subjects; therefore, ethical approval was not required.

## The hypoxic tumor microenvironment in breast cancer

The tumor microenvironment (TME) in breast cancer is a complex and dynamic ecosystem composed of malignant cells, stromal components, immune cells, and an aberrant vasculature. As the tumor mass expands, the demand for oxygen surpasses the supply provided by the existing blood vessels, leading to the development of hypoxic regions. Hypoxia within the tumor is not uniform; instead, it exists in gradients ranging from mild to severe oxygen deprivation, depending on the distance of cells from functional blood vessels. These hypoxic zones are not merely passive byproducts of tumor growth but are active drivers of malignancy, influencing a wide array of cellular processes and contributing to tumor heterogeneity (15, 16). The primary cellular response to hypoxia is mediated by the hypoxia-inducible factors (HIFs), which function as oxygen-sensitive transcription factors. Under normoxic conditions, HIF-1α is hydroxylated by prolyl hydroxylases, leading to its ubiquitination and subsequent degradation via the von Hippel-Lindau (VHL) tumor suppressor protein. In hypoxic conditions, this hydroxylation process is inhibited, allowing HIF-1α to accumulate, dimerize with HIF-1β, and translocate to the nucleus. There, it activates the transcription of genes involved in critical survival pathways, including angiogenesis (e.g., VEGF), glycolysis (e.g., GLUT1, LDHA), erythropoiesis, and cell proliferation (17, 18).

In breast cancer, chronic hypoxia exerts selective pressure that favors the survival and expansion of more aggressive phenotypes. It has been linked to enhanced epithelial-mesenchymal transition (EMT), a key process in metastasis, as well as increased resistance to therapy. Furthermore, hypoxic regions tend to be immunosuppressive, characterized by the recruitment of regulatory T cells and myeloid-derived suppressor cells, as well as the inhibition of cytotoxic T cell function. This immunosuppressive environment facilitates immune evasion, enabling tumor progression (19, 20). Importantly, the vasculature in hypoxic breast tumors is often disorganized and leaky, contributing to erratic blood flow and further aggravating oxygen deficiency. This chaotic vascular architecture results in repeated cycles of acute and chronic hypoxia, which further promote genetic instability and intratumoral heterogeneity. Additionally, the acidic environment resulting from anaerobic metabolism disrupts normal cell-cell and cell-matrix interactions, enhancing invasion and migration (21, 22). The clinical implications of hypoxia in breast cancer are profound. Studies have shown that hypoxia-related gene signatures are associated with poor prognosis, higher tumor grade, and increased likelihood of metastasis. Moreover, hypoxia reduces the efficacy of radiotherapy, which relies on the presence of oxygen to generate reactive oxygen species that damage DNA. Similarly, many chemotherapeutic agents are less effective in hypoxic environments due to altered drug metabolism and reduced proliferation of hypoxic cells (Figure 1) (23).

**FIGURE 1:**
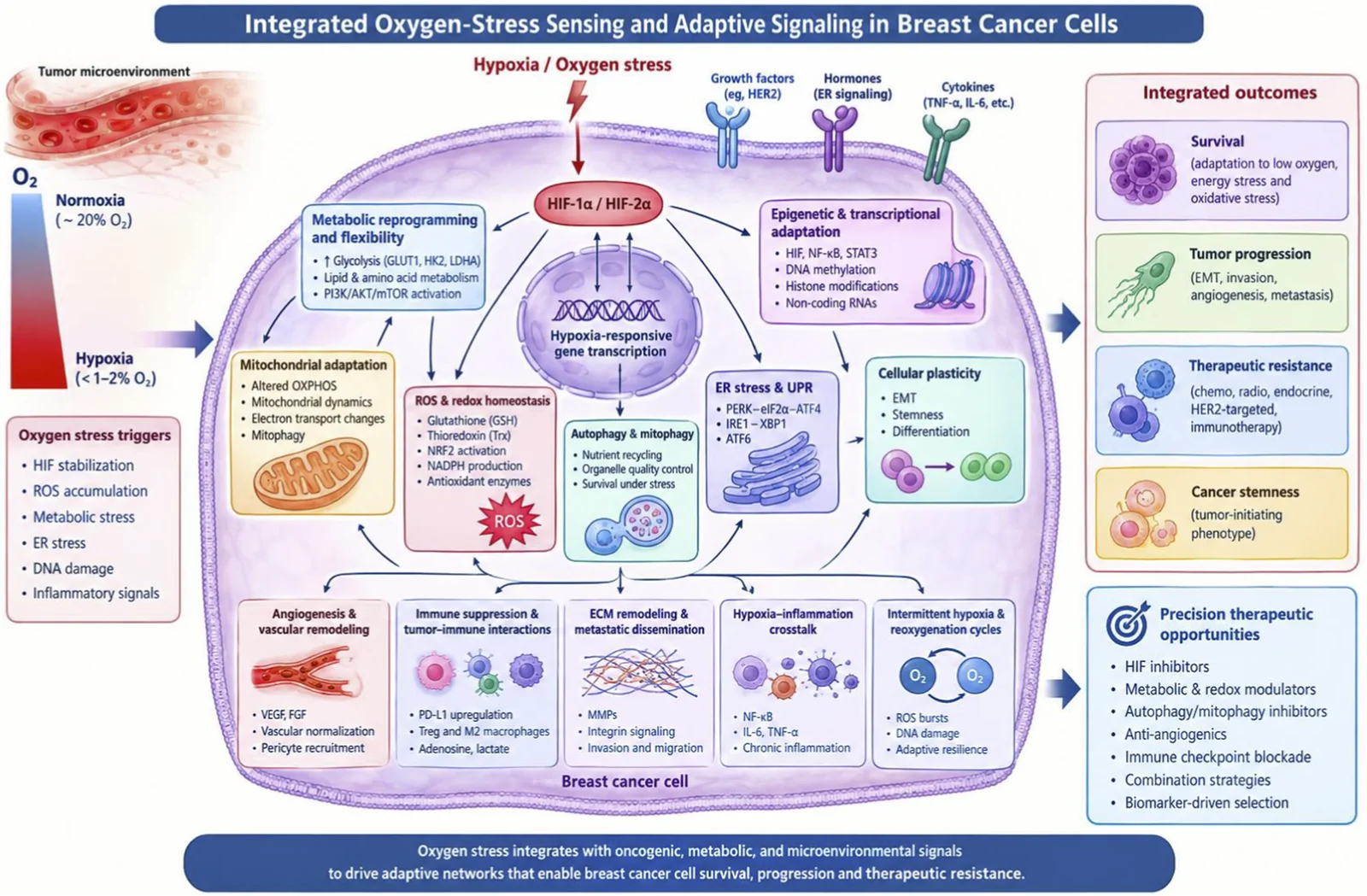
Integrated oxygen-stress sensing and adaptive signaling in breast cancer cells.

### Metabolic reprogramming in response to hypoxia in breast cancer

Metabolic reprogramming is a hallmark of cancer, enabling tumor cells to meet the increased energetic and biosynthetic demands of rapid proliferation. In the context of hypoxia, this metabolic plasticity becomes even more pronounced. Oxygen deprivation forces breast cancer cells to shift their energy production from mitochondrial oxidative phosphorylation to glycolysis, even when oxygen is partially available. This phenomenon, known as the Warburg effect, is amplified under hypoxic conditions and is orchestrated primarily by hypoxia-inducible factors (HIFs), especially HIF-1α (24–26). Under normal physiological conditions, cells rely on oxidative phosphorylation in mitochondria to generate adenosine triphosphate (ATP) efficiently. However, this process requires oxygen as a terminal electron acceptor in the electron transport chain. In hypoxic tumor regions, where oxygen is limited or absent, oxidative phosphorylation is compromised. HIF-1α compensates by upregulating the expression of glycolytic enzymes such as hexokinase 2 (HK2), phosphofructokinase (PFK), and lactate dehydrogenase A (LDHA). These enzymes enhance the glycolytic flux, allowing cells to generate ATP through substrate-level phosphorylation despite the reduced efficiency of the pathway (12, 27, 28).

Additionally, hypoxia induces the expression of glucose transporters, particularly GLUT1 and GLUT3, to increase glucose uptake from the extracellular environment. This adaptation ensures a continuous supply of glucose to fuel glycolysis. The end product of anaerobic glycolysis, lactate, accumulates in the tumor microenvironment, contributing to acidosis. This acidic milieu further supports tumor progression by promoting extracellular matrix degradation, angiogenesis, and immune evasion. Some breast cancer cells also express monocarboxylate transporters (MCTs), which export lactate out of the cell, helping to maintain intracellular pH homeostasis and sustain glycolytic activity (15, 29, 30). Beyond glycolysis, hypoxic breast cancer cells exhibit alterations in other metabolic pathways, including glutaminolysis and lipid metabolism. Glutamine becomes an alternative carbon source for the tricarboxylic acid (TCA) cycle, supporting biosynthesis and redox balance. Meanwhile, lipid synthesis and storage are modulated to meet the demands for membrane biosynthesis and to buffer oxidative stress. These metabolic changes are not merely passive responses to environmental stress but are actively regulated to support survival, proliferation, and resistance to therapy (31–33). Importantly, this hypoxia-driven metabolic rewiring provides cancer cells with a selective advantage in the tumor microenvironment. It supports not only growth under low oxygen but also confers resistance to therapies that target rapidly dividing, oxygen-dependent cells. Moreover, metabolic flexibility allows hypoxic breast cancer cells to survive nutrient fluctuations and to outcompete less-adapted neighboring cells. Consequently, metabolic reprogramming contributes to intratumoral heterogeneity and the emergence of more aggressive subpopulations (Table 1) (34, 35).

**TABLE 1:** Metabolic reprogramming in response to hypoxia in breast cancer.

Metabolic pathway	Hypoxia-induced change	Key regulatory mechanisms	Functional consequence in breast cancer cells	Clinical/Pathological implication
Glycolysis (Warburg shift)	Increased reliance on anaerobic glycolysis even in oxygen presence	HIF-1α upregulation of GLUT1, HK2, PFK, LDHA	Enhanced ATP production independent of mitochondria; lactate accumulation	Tumor acidification, invasion, and poor prognosis
Oxidative phosphorylation (OXPHOS)	Suppressed mitochondrial respiration	HIF-mediated inhibition of mitochondrial biogenesis (via PGC-1α suppression)	Reduced oxygen consumption and mitochondrial efficiency	Therapy resistance and metabolic plasticity
Glucose uptake	Increased glucose uptake	Upregulation of GLUT1 and GLUT3 transporters	Enhanced substrate availability for glycolysis	Increased FDG-PET signal in hypoxic tumors
Lactate metabolism	Increased lactate production and export	Upregulation of LDHA and MCT4 transporters	Acidic tumor microenvironment	Immune suppression and enhanced metastasis
Pentose phosphate pathway (PPP)	Enhanced flux through PPP	HIF-driven activation of G6PD	Increased NADPH and ribose-5-phosphate production	Redox balance maintenance and nucleotide synthesis
Fatty acid metabolism	Shift toward fatty acid uptake and oxidation in adaptive clones	AMPK activation and lipid transport proteins (CD36)	Energy production under nutrient stress	Supports metastatic and stem-like phenotypes
Glutamine metabolism	Increased glutamine dependency	c-Myc and HIF interaction regulating glutaminase (GLS)	Anaplerosis for TCA cycle intermediates	Supports biosynthesis and survival under hypoxia
Mitochondrial ROS production	Increased but tightly regulated ROS levels	HIF signaling and antioxidant response (Nrf2 activation)	Redox signaling for survival and adaptation	Promotes DNA damage tolerance and tumor evolution
Autophagy	Upregulated autophagic flux	HIF-1α → BNIP3/BNIP3L activation	Recycling of cellular components for energy	Enhances survival in nutrient- and oxygen-deprived regions
pH regulation	Increased acid extrusion	Carbonic anhydrase IX (CAIX) and proton transporters	Maintenance of intracellular pH stability	Promotes invasion and metastatic potential
Metabolic heterogeneity	Coexistence of glycolytic and oxidative tumor cell populations	Microenvironmental oxygen gradients	Division of metabolic labor within tumor microenvironment	Therapy resistance and tumor adaptability

Abbreviations: AMPK, AMP-activated protein kinase; CAIX, carbonic anhydrase IX; FDG-PET, fluorodeoxyglucose positron emission tomography; GLS, glutaminase; GLUT, glucose transporter; HIF-1α, hypoxia-inducible factor 1-alpha; LDHA, lactate dehydrogenase A; MCT4, monocarboxylate transporter 4; OXPHOS, oxidative phosphorylation; PFK, phosphofructokinase; PPP, pentose phosphate pathway; ROS, reactive oxygen species.

### Angiogenesis and vascular remodeling in breast cancer

Angiogenesis—the formation of new blood vessels from pre-existing vasculature—is a critical adaptation that allows breast cancer cells to survive and grow in hypoxic conditions. As tumors expand beyond a certain size, diffusion becomes insufficient to deliver adequate oxygen and nutrients, leading to cellular stress. To overcome this, hypoxia triggers a well-coordinated angiogenic response primarily driven by the hypoxia-inducible factor (HIF) pathway, especially through the upregulation of vascular endothelial growth factor (VEGF). This response enables the tumor to recruit and remodel vasculature, improving perfusion and sustaining its growth (36, 37). HIF-1α plays a central role in activating the angiogenic switch under low-oxygen conditions. In breast cancer, HIF-1α stabilization leads to increased transcription of VEGF and other pro-angiogenic mediators, such as platelet-derived growth factor (PDGF), angiopoietins, and stromal-derived factor 1 (SDF-1). VEGF is a potent mitogen for endothelial cells, stimulating their proliferation, migration, and differentiation into new capillary structures. These new blood vessels aim to re-establish oxygen and nutrient delivery to the hypoxic tumor zones. However, in the context of malignancy, the angiogenesis process is often dysregulated (38, 39).

Unlike the organized vasculature of normal tissues, tumor-associated blood vessels in breast cancer are typically abnormal—characterized by tortuosity, leakiness, irregular diameter, and poor pericyte coverage. This aberrant vascular architecture results in uneven and inefficient blood flow, leading to areas of continued or intermittent hypoxia. Such regions create cycles of acute and chronic hypoxia, which further exacerbate tumor heterogeneity and foster a selection pressure for more aggressive cancer cell phenotypes. Moreover, the leaky nature of tumor vasculature facilitates tumor cell intravasation into the bloodstream, promoting metastasis (40, 41). Vascular remodeling in breast cancer involves not only the formation of new vessels but also the alteration of existing vascular networks. Hypoxia stimulates the recruitment of endothelial progenitor cells (EPCs) from the bone marrow, contributing to neovascularization. Additionally, tumor-associated macrophages (TAMs) and cancer-associated fibroblasts (CAFs) within the tumor microenvironment secrete angiogenic factors that amplify vascular growth and remodeling. This crosstalk between cancer cells and stromal components is essential for sustaining the pro-angiogenic state and maintaining tumor vascular plasticity (42).

Angiogenesis also interacts with other hypoxia-driven processes such as metabolism, immune modulation, and extracellular matrix remodeling. For instance, the acidic microenvironment created by lactate accumulation can enhance VEGF production and endothelial cell migration. Moreover, VEGF itself exerts immunosuppressive effects by inhibiting dendritic cell maturation and promoting the expansion of regulatory T cells, further contributing to immune evasion in the hypoxic tumor niche (43). Targeting angiogenesis has been a focus of therapeutic strategies in breast cancer. Anti-VEGF agents, such as bevacizumab, have shown efficacy in reducing tumor vascularization and improving progression-free survival in some breast cancer subtypes. However, clinical benefits have often been transient and modest, partly due to compensatory angiogenic pathways and the persistence of hypoxia. Additionally, excessive pruning of blood vessels can paradoxically worsen hypoxia, enhancing tumor aggressiveness and therapeutic resistance (Table 2) (44).

**TABLE 2:** Angiogenesis and vascular remodeling in breast cancer under oxygen stress.

Component	Hypoxia-induced change	Key molecular drivers	Cellular/Structural effect	Clinical consequence
VEGF signaling pathway	Strong upregulation of angiogenic signaling	HIF-1α–mediated VEGF-A transcription	Increased endothelial cell proliferation and new vessel formation	Tumor growth acceleration and poor prognosis
VEGF receptors (VEGFR-1/2)	Enhanced receptor expression on endothelial cells	VEGF ligand binding and downstream MAPK/PI3K activation	Increased angiogenic responsiveness	Promotes vascular expansion in hypoxic regions
Abnormal vessel formation	Disorganized, tortuous, and leaky vasculature	Imbalanced pro- and anti-angiogenic signaling	Poorly structured and inefficient blood vessels	Persistent tumor hypoxia and drug delivery impairment
Endothelial cell activation	Increased proliferation and migration	VEGF, ANGPT2, Notch signaling	Endothelial sprouting and unstable vascular networks	Facilitates tumor perfusion heterogeneity
Angiopoietin signaling (ANGPT1/2–TIE2 axis)	ANGPT2 upregulation promotes vascular instability	Hypoxia-driven transcriptional regulation	Vessel destabilization and remodeling	Enhances metastatic potential
Pericyte detachment	Reduced pericyte coverage of vessels	PDGF-B downregulation and VEGF imbalance	Weak vascular support and increased permeability	Promotes tumor cell intravasation and metastasis
Hypoxia-driven vascular remodeling	Cyclical vessel collapse and regrowth	HIF signaling and metabolic stress responses	Alternating perfusion and hypoxia cycles	Selection of aggressive, therapy-resistant clones
Lymphangiogenesis	Increased lymphatic vessel formation	VEGF-C and VEGF-D upregulation	Expansion of lymphatic network	Enhanced lymph node metastasis
Extracellular matrix (ECM) remodeling	Increased matrix degradation and stiffness changes	Matrix metalloproteinases (MMP-2, MMP-9)	Facilitates endothelial and tumor cell migration	Promotes invasion and metastatic dissemination
Tumor vascular normalization (therapeutic target)	Partial restoration of vessel structure under therapy	Anti-VEGF agents (e.g., bevacizumab)	Improved perfusion and oxygenation (temporary)	Enhanced chemotherapy/radiotherapy delivery
Vascular permeability increase	Leaky endothelial junctions	VE-cadherin disruption and VEGF signaling	Extravasation of plasma proteins and tumor cells	Contributes to edema and metastatic spread

Abbreviations: ANGPT, angiopoietin; ECM, extracellular matrix; HIF-1α, hypoxia-inducible factor 1-alpha; MAPK, mitogen-activated protein kinase; MMP, matrix metalloproteinase; PDGF-B, platelet-derived growth factor B; PI3K, phosphoinositide 3-kinase; TIE2, tyrosine kinase with immunoglobulin-like and EGF-like domains; VEGF, vascular endothelial growth factor; VEGFR, vascular endothelial growth factor receptor.

### Evasion of apoptosis and enhanced survival in breast cancer

In healthy tissues, cells experiencing severe stress—such as nutrient deprivation, DNA damage, or hypoxia—typically undergo programmed cell death, or apoptosis, as a protective mechanism to maintain tissue integrity. However, breast cancer cells exposed to hypoxic conditions often evade apoptosis through a range of adaptive mechanisms that promote survival and continued tumor progression. These adaptations are not incidental but are actively regulated through both transcriptional and post-translational modifications, largely orchestrated by hypoxia-inducible factors (HIFs), particularly HIF-1α (45, 46). Under hypoxic stress, HIF-1α alters the balance of pro-apoptotic and anti-apoptotic signaling pathways to favor cell survival. One key adaptation involves the upregulation of anti-apoptotic proteins such as B-cell lymphoma 2 (BCL-2), BCL-XL, and survivin. These proteins inhibit the mitochondrial pathway of apoptosis by preventing cytochrome c release and caspase activation, effectively blocking cell death. At the same time, hypoxia can suppress the expression or activity of pro-apoptotic factors like BAX, PUMA, and BID, further tipping the balance toward survival. This altered apoptotic threshold allows breast cancer cells to persist and even thrive in hostile microenvironments that would be lethal to normal cells (47–49).

Autophagy, a self-digestive cellular process, also plays a critical role in the survival of hypoxic breast cancer cells. When oxygen and nutrient availability are limited, autophagy is upregulated as a protective mechanism to recycle cellular components and maintain metabolic homeostasis. HIF-1α and AMP-activated protein kinase (AMPK) pathways contribute to this process by inducing genes involved in autophagosome formation and lysosomal degradation. While autophagy can act as a form of programmed cell death under certain conditions, in hypoxic breast tumors it is more commonly associated with cytoprotective functions that support cell viability and resistance to therapy (50–52). Additionally, breast cancer cells under hypoxia often exhibit alterations in the p53 signaling pathway. The tumor suppressor protein p53 is a major regulator of apoptosis and cellular stress responses. In many breast cancers, p53 is mutated or functionally inactivated, allowing cancer cells to bypass p53-dependent apoptotic mechanisms. Even in tumors with wild-type p53, hypoxia can impair its transcriptional activity, reducing the expression of downstream pro-apoptotic targets. This impairment of p53 function under hypoxic conditions represents another critical node of apoptosis evasion (53–55).

The hypoxic microenvironment also fosters the development of cancer stem-like cells (CSCs), which possess enhanced survival capabilities and are more resistant to apoptosis. These CSCs are thought to be responsible for tumor recurrence and metastasis, in part due to their quiescent nature and heightened expression of survival proteins. Hypoxia supports the maintenance and self-renewal of these cells through HIF-mediated signaling, including activation of Notch, Wnt, and Hedgehog pathways—all of which are associated with stemness and survival (56, 57). The ability of breast cancer cells to evade apoptosis under hypoxic conditions contributes significantly to treatment resistance. Radiotherapy and many forms of chemotherapy rely on the induction of DNA damage and subsequent apoptotic cell death. Hypoxic cells, with their suppressed apoptotic machinery and enhanced repair capabilities, are less responsive to these interventions. This underscores the need for therapeutic strategies that can restore apoptotic sensitivity or exploit alternative forms of cell death in hypoxic tumor cells (Table 3) (58).

**TABLE 3:** Evasion of apoptosis and enhanced survival in breast cancer under oxygen stress.

Apoptotic/Survival pathway	Hypoxia-induced change	Key molecular regulators	Functional effect in breast cancer cells	Clinical/Oncologic implication
Intrinsic (mitochondrial) apoptosis pathway	Suppression of mitochondrial apoptosis signaling	HIF-1α, BCL-2 upregulation, BAX downregulation	Stabilization of mitochondrial membrane integrity	Reduced chemotherapy-induced cell death
Extrinsic (death receptor) pathway	Reduced sensitivity to death ligands	Downregulation of Fas/FasL and TRAIL receptors	Impaired extrinsic apoptosis activation	Immune evasion and therapy resistance
BCL-2 family balance	Shift toward anti-apoptotic dominance	Increased BCL-2, BCL-XL; decreased BAX/BAK	Inhibition of cytochrome c release	Enhanced tumor cell survival under stress
p53 tumor suppressor pathway	Functional suppression or mutation-driven loss	Hypoxia-associated MDM2 activation, p53 destabilization	Impaired DNA damage response and apoptosis initiation	Increased genomic instability and tumor progression
PI3K/AKT signaling	Pathway hyperactivation	HIF-mediated activation of PI3K/AKT/mTOR axis	Promotion of cell survival and proliferation	Resistance to endocrine and targeted therapies
Autophagy activation	Increased autophagic flux as survival mechanism	HIF-1α → BNIP3/BNIP3L signaling	Recycling of cellular components for energy production	Supports survival in nutrient- and oxygen-deprived regions
Endoplasmic reticulum (ER) stress response	Adaptive unfolded protein response activation	PERK, ATF6, IRE1 pathways	Restoration of protein-folding homeostasis	Enhances survival under metabolic stress
Reactive oxygen species (ROS) regulation	Controlled ROS accumulation	Nrf2 pathway activation, antioxidant enzyme upregulation	Maintenance of redox balance for survival signaling	Promotes resistance to oxidative damage and therapy
Cell cycle arrest adaptation	Reversible G0/G1 or G2/M arrest under hypoxia	HIF-mediated regulation of cyclins and CDKs	Allows DNA repair and survival under stress	Contributes to treatment resistance and tumor dormancy
Cancer stem cell enrichment	Increased stem-like phenotype population	HIF-1α, Notch, Wnt/β-catenin signaling	Enhanced self-renewal and tumor initiation capacity	Associated with relapse and metastasis
Immune evasion synergy	Reduced immunogenicity under hypoxia	PD-L1 upregulation, cytokine modulation	Reduced cytotoxic T-cell activity	Resistance to immunotherapy and immune clearance

Abbreviations: AKT, protein kinase B; ATF6, activating transcription factor 6; BAX, BCL-2-associated X protein; BCL-2, B-cell lymphoma 2; BNIP3, BCL2/adenovirus E1B 19 kDa protein-interacting protein 3; CDKs, cyclin-dependent kinases; ER, endoplasmic reticulum; Fas/FasL, fas receptor and ligand; HIF-1α, hypoxia-inducible factor 1-alpha; IRE1, inositol-requiring enzyme 1; MDM2, mouse double minute 2 homolog; mTOR, mechanistic target of rapamycin; Nrf2, nuclear factor erythroid 2–related factor 2; PI3K, phosphoinositide 3-kinase; PERK, protein kinase R-like ER, kinase; TRAIL, TNF-related apoptosis-inducing ligand.

### Mitochondrial adaptation: oxidative phosphorylation, electron transport, dynamics, and mitophagy

Although hypoxia is traditionally associated with suppression of mitochondrial oxidative phosphorylation, mitochondria remain critical regulators of breast cancer adaptation to oxygen stress. Hypoxic tumor cells do not simply switch mitochondria off; instead, they remodel mitochondrial activity to reduce oxygen consumption, maintain bioenergetic stability, control ROS production, and preserve cellular viability. HIF-1α contributes to this process by suppressing mitochondrial oxygen consumption and redirecting pyruvate away from oxidative metabolism. Regulation of pyruvate dehydrogenase through pyruvate dehydrogenase kinase reduces entry of pyruvate into the tricarboxylic acid cycle. This metabolic adjustment conserves oxygen and decreases mitochondrial electron flow under oxygen-limited conditions. Nevertheless, the electron transport chain remains active to varying degrees. Partial oxygen reduction at mitochondrial complexes can generate ROS, particularly during transitions between oxygen availability states. Rather than functioning exclusively as toxic molecules, mitochondrial ROS can act as signaling intermediates that reinforce HIF stabilization and activate stress-adaptive pathways. Excessive ROS, however, causes oxidative damage to mitochondrial DNA, proteins, and membrane lipids (59, 60).

Mitochondrial dynamics also contribute to adaptation. The balance between mitochondrial fusion and fission determines mitochondrial morphology, distribution, bioenergetic capacity, and susceptibility to quality-control mechanisms. Hypoxic stress can alter this balance and promote selective removal of dysfunctional mitochondria. Mitochondrial fission can facilitate segregation of damaged organelles, while fusion may support functional complementation between mitochondria. Mitophagy provides another critical layer of mitochondrial adaptation. Selective autophagic elimination of damaged mitochondria prevents excessive ROS accumulation and preserves mitochondrial quality. HIF-dependent and HIF-independent mechanisms can contribute to mitophagy under hypoxic conditions. In breast cancer, this may allow tumor cells to tolerate prolonged metabolic stress while preventing mitochondrial damage from reaching a lethal threshold. Mitochondria also influence apoptosis. Mitochondrial outer-membrane permeabilization is a major determinant of intrinsic apoptotic signaling, and hypoxic adaptation can modify the balance between pro- and anti-apoptotic proteins. Increased expression of survival factors and altered mitochondrial metabolism can therefore make oxygen-stressed tumor cells less responsive to cytotoxic therapy (61, 62).

### ROS generation and redox homeostasis

Oxygen stress creates a paradox for cancer cells: oxygen deprivation reduces oxygen-dependent metabolism, yet hypoxic and especially reoxygenated cells can experience substantial oxidative stress. Breast cancer cells therefore require highly coordinated redox systems to maintain ROS within a range that supports signaling without causing lethal damage. Mitochondrial electron transport is an important source of ROS during oxygen fluctuations. In addition, NADPH oxidases, peroxisomal metabolism, inflammatory signaling, and reoxygenation can contribute to ROS generation. Moderate ROS concentrations can activate signaling pathways involved in proliferation, migration, HIF stabilization, and stress adaptation. Excessive ROS, however, can induce lipid peroxidation, protein oxidation, DNA damage, mitochondrial dysfunction, and cell death. Breast cancer cells adapt by increasing antioxidant capacity. The glutathione system is particularly important. Reduced glutathione acts as a major intracellular antioxidant and participates in detoxification of reactive oxygen species and electrophilic compounds. Maintenance of the reduced glutathione pool depends on adequate NADPH availability (63, 64).

The thioredoxin system provides another major redox-control mechanism. Thioredoxin proteins maintain protein thiol groups in a reduced state and support cellular resistance to oxidative injury. Hypoxia-adapted tumor cells may therefore become unusually dependent on thioredoxin-mediated redox regulation. The transcription factor NRF2 is a central regulator of antioxidant gene expression. Activation of NRF2 can increase expression of enzymes involved in glutathione synthesis, detoxification, NADPH generation, and ROS removal. Crosstalk between NRF2 and oncogenic signaling can reinforce tumor survival under metabolic and therapeutic stress. NADPH represents a critical metabolic currency linking glucose metabolism, lipid metabolism, and redox defense. The pentose phosphate pathway, malic enzyme activity, and other metabolic processes contribute to NADPH production. Hypoxic tumor cells can therefore redirect carbon flux toward pathways that preserve reducing equivalents rather than maximize ATP production alone (Figure 2) (65, 66).

**FIGURE 2:**
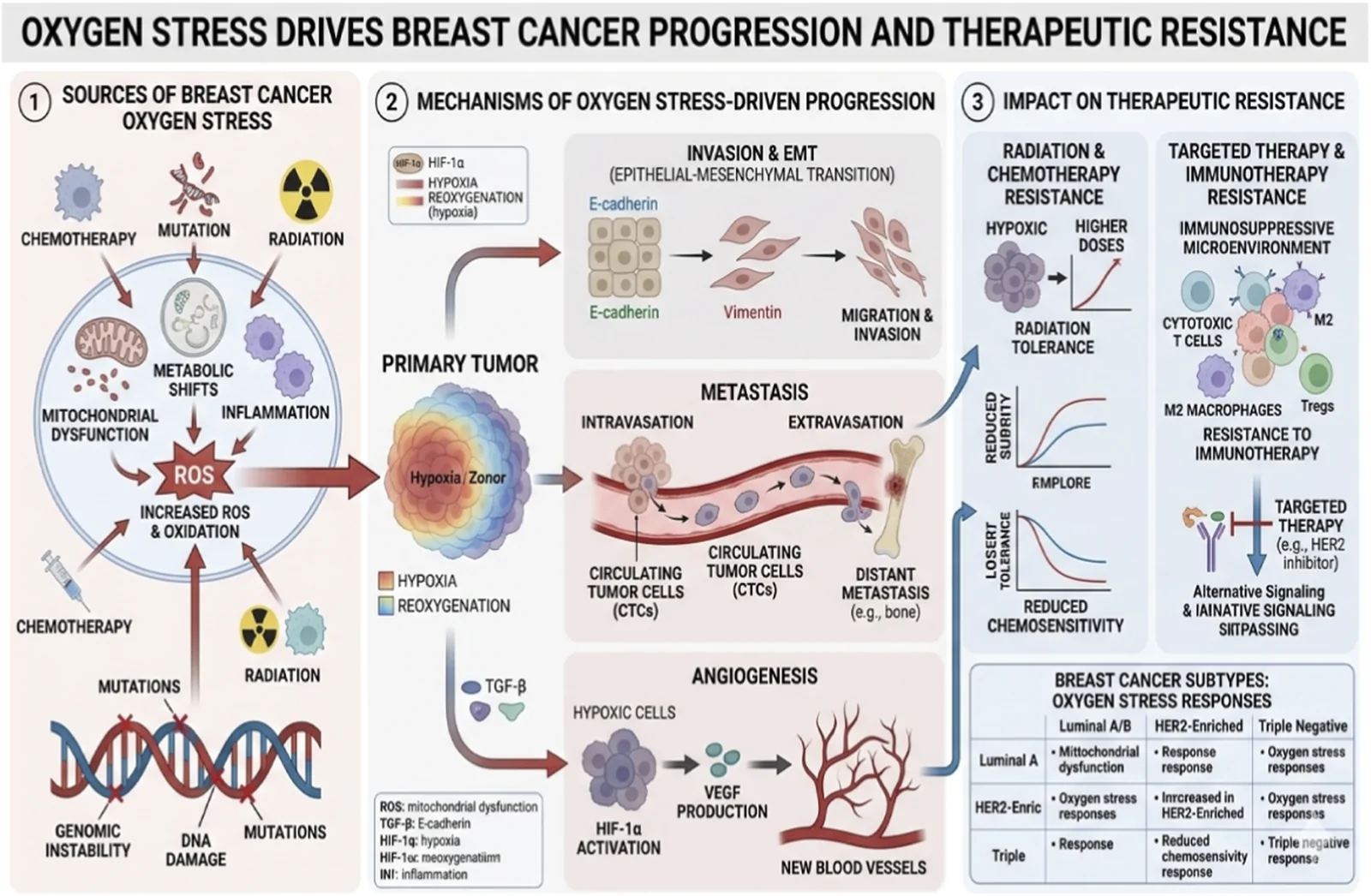
Oxygen stress as a driver of breast cancer progression and therapeutic resistance.

### Autophagy and mitophagy as survival mechanisms

Autophagy is an essential cellular quality-control process that becomes increasingly important when breast cancer cells encounter hypoxia, nutrient deprivation, metabolic stress, or anticancer therapy. By degrading damaged proteins and organelles and recycling their constituent molecules, autophagy allows cells to maintain energy balance and cellular integrity under adverse conditions. Hypoxia can activate autophagy through HIF-dependent and energy-sensing pathways. AMPK activation during energetic stress can stimulate autophagy, while suppression of mTOR signaling removes an important inhibitory influence on autophagic activity. Autophagy can therefore provide a survival mechanism during periods when nutrient and oxygen availability are inadequate. Mitophagy is particularly important because damaged mitochondria can become major sources of ROS. Selective elimination of dysfunctional mitochondria prevents excessive oxidative injury while allowing healthier mitochondria to maintain residual metabolic activity. In this way, mitophagy links mitochondrial quality control with redox homeostasis. Autophagy may also contribute to therapeutic resistance. Chemotherapy and radiotherapy impose metabolic and oxidative stress, potentially stimulating autophagic recycling in surviving tumor cells. These cells may subsequently recover and repopulate the tumor. Nevertheless, autophagy is biologically context-dependent. Excessive or dysregulated autophagy can contribute to cell death, and autophagy may have tumor-suppressive functions in some stages of tumor development. Consequently, clinical application of autophagy inhibitors requires identification of tumors in which autophagy is functioning predominantly as a survival mechanism (67, 68).

### Endoplasmic reticulum stress and the unfolded protein response

Hypoxia disrupts protein folding, calcium homeostasis, energy metabolism, and cellular redox balance, thereby imposing stress on the endoplasmic reticulum (ER). Breast cancer cells respond through the unfolded protein response (UPR), a coordinated signaling network designed initially to restore proteostasis. The three major UPR sensors are PERK, IRE1, and ATF6. PERK reduces global protein translation while promoting selective expression of stress-response proteins. IRE1 regulates stress-responsive transcription and RNA processing, whereas ATF6 increases expression of molecular chaperones and other components required for protein-folding capacity. During moderate hypoxia, the UPR can promote survival by reducing metabolic demands and preventing accumulation of misfolded proteins. However, prolonged or severe ER stress may activate apoptosis. The balance between adaptive and terminal UPR signaling is therefore a critical determinant of whether oxygen-stressed breast cancer cells survive. The UPR also interacts with HIF signaling, autophagy, metabolism, and inflammatory pathways. This creates a highly integrated stress-response network. Therapeutically, inhibition of adaptive UPR signaling could sensitize hypoxic breast cancer cells to chemotherapy or metabolic stress, particularly in tumors demonstrating strong proteostasis dependence (69, 70).

### Hypoxia-induced EMT and cellular plasticity

Hypoxia is a potent regulator of breast cancer cellular plasticity. Through HIF-dependent and HIF-independent mechanisms, oxygen stress can promote epithelial–mesenchymal transition (EMT), enabling epithelial tumor cells to acquire migratory, invasive, and therapy-resistant characteristics. HIF signaling interacts with EMT transcription factors including SNAIL, SLUG, TWIST, and ZEB family proteins. These factors suppress epithelial differentiation programs, including E-cadherin expression, while promoting mesenchymal phenotypes. The consequence is increased motility, extracellular matrix interaction, invasion, and dissemination. Importantly, EMT is not necessarily a complete binary transition. Breast cancer cells may occupy intermediate or hybrid epithelial/mesenchymal states. These states can combine epithelial proliferative capacity with mesenchymal migratory properties. Cellular plasticity also facilitates adaptation to therapy. Treatment may eliminate highly proliferative tumor cells while leaving slower-cycling, stress-adapted populations capable of later re-entering proliferative states. Consequently, targeting plasticity may require strategies that prevent transitions between cellular states rather than eliminating one fixed phenotype (71, 72).

### Cancer stemness and tumor-initiating phenotypes

Hypoxic niches can promote or preserve breast cancer stem-like populations. HIF signaling interacts with developmental pathways such as Notch, Wnt/β-catenin, Hedgehog, and TGF-β to regulate self-renewal and cellular differentiation. Cancer stem-like cells possess several properties that are particularly advantageous under oxygen stress. They may demonstrate increased antioxidant capacity, enhanced DNA repair, altered metabolism, reduced apoptosis, and increased drug-efflux activity. Hypoxia may therefore function as a selective force that enriches for tumor cells capable of surviving treatment and subsequently regenerating heterogeneous tumor populations. This provides a mechanistic connection between oxygen stress and disease relapse. A therapy may produce substantial tumor shrinkage while failing to eliminate hypoxia-adapted tumor-initiating populations. Targeting stemness-associated signaling in combination with oxygen-stress-directed therapy may therefore be necessary to achieve durable tumor control (73, 74).

### Hypoxia-associated immune suppression and tumor–immune interactions

Oxygen stress profoundly influences the breast cancer immune microenvironment. Hypoxic tumor regions can become immunologically dysfunctional through several complementary mechanisms. Hypoxia can impair cytotoxic T-cell and natural killer-cell activity while promoting immunosuppressive cellular populations. Tumor-associated macrophages can acquire phenotypes that support angiogenesis, tissue remodeling, and tumor progression. Hypoxia also increases extracellular adenosine, which can suppress immune effector function through purinergic signaling. HIF signaling can influence immune checkpoint expression and cytokine production, while metabolic competition between tumor cells and immune cells further restricts immune activity. High lactate concentrations and acidic extracellular conditions can impair T-cell function and favor immunosuppressive phenotypes. These mechanisms may help explain why a tumor containing immune cells may nevertheless remain functionally immune resistant. Hypoxia-directed therapy could therefore potentially enhance immunotherapy by improving immune-cell function, vascular access, and antigen-directed activity. Rational combinations of hypoxia modulation with immune checkpoint inhibition represent an important translational opportunity (75, 76).

### Extracellular matrix remodeling and metastatic dissemination

Hypoxia promotes extensive remodeling of the extracellular matrix (ECM), transforming the physical and biochemical environment surrounding breast cancer cells. HIF-regulated factors can increase expression of matrix-remodeling enzymes, alter collagen organization, and modify cell–matrix interactions. These changes can increase tissue stiffness, facilitate invasion, and create migration pathways. Hypoxia also interacts with cancer-associated fibroblasts and other stromal cells. These cells can produce extracellular matrix components, growth factors, and cytokines that reinforce tumor adaptation. At the invasive tumor margin, hypoxia-associated EMT, matrix remodeling, angiogenesis, and protease activity can converge to facilitate tumor-cell dissemination. This mechanism links local oxygen stress with systemic metastasis and suggests that the hypoxic microenvironment should be considered a potential regulator of the metastatic cascade rather than merely a feature of the primary tumor (77, 78).

### Hypoxia–inflammation crosstalk

Hypoxia and inflammation form a reciprocal signaling network within breast tumors. HIF signaling interacts extensively with NF-κB, STAT3, inflammatory cytokines, and other stress pathways. Inflammatory mediators can enhance HIF activity, while hypoxia can stimulate production of cytokines and chemokines that recruit and reprogram stromal and immune cells. This reciprocal interaction generates a feed-forward system that supports angiogenesis, invasion, metabolic adaptation, and immune suppression. Tumor-associated macrophages are particularly important intermediaries. Hypoxic areas can recruit macrophages and promote their differentiation toward tumor-supportive phenotypes. These macrophages, in turn, produce angiogenic and inflammatory mediators that further reinforce hypoxic adaptation. Consequently, targeting hypoxia without addressing inflammatory signaling may permit continued microenvironmental support of tumor survival (79, 80).

### Breast cancer subtype-specific responses to oxygen stress

Breast cancer is a biologically heterogeneous disease, and the consequences of oxygen stress are strongly influenced by molecular subtype, oncogenic signaling, metabolic phenotype, receptor status, and the composition of the tumor microenvironment. Although HIF-mediated adaptation represents a common response to hypoxia across breast cancers, the downstream consequences are not uniform. Hormone receptor-positive (HR-positive), HER2-positive, and triple-negative breast cancers (TNBC) can differ substantially in their dependence on glycolysis, mitochondrial metabolism, redox regulation, epithelial–mesenchymal plasticity, immune signaling, and stress-response pathways. Consequently, oxygen stress should be regarded as a subtype-contextual biological pressure rather than a universal molecular phenotype.

### Hormone receptor-positive breast cancer

Hormone receptor-positive breast cancers, particularly estrogen receptor-positive (ER-positive) tumors, demonstrate complex interactions between oxygen sensing and hormone-dependent transcription. Hypoxia can influence estrogen receptor signaling through HIF-dependent transcriptional remodeling and activation of alternative growth and survival pathways. HIF-1α and estrogen receptor signaling can interact at multiple levels, potentially allowing tumor cells to maintain proliferation when oxygen availability is restricted or estrogen signaling is therapeutically suppressed. Oxygen stress may also promote metabolic adaptation in ER-positive disease. Although many HR-positive tumors retain substantial mitochondrial oxidative capacity, hypoxia can increase glycolytic flux and alter utilization of lipids and amino acids. This flexibility may permit survival during endocrine therapy by reducing dependence on a single metabolic or receptor-mediated growth pathway. Activation of PI3K/AKT/mTOR signaling provides an additional route through which hypoxic cells can maintain anabolic activity and survival despite therapeutic suppression of estrogen signaling. Hypoxia may be particularly relevant to endocrine resistance. HIF-mediated transcription can promote estrogen-independent survival programs, while activation of growth-factor and PI3K/AKT/mTOR pathways can bypass receptor blockade. Hypoxia-associated EMT and cellular plasticity may further generate tumor-cell states with reduced dependence on ER signaling. These mechanisms suggest that oxygen stress can contribute not only to initial treatment resistance but also to the evolution of acquired endocrine-resistant populations. However, HR-positive breast cancers are heterogeneous. Luminal A and luminal B tumors may differ in proliferation, metabolic activity, genomic alterations, and hypoxia-associated transcriptional responses. Therefore, hypoxia-targeted approaches should ideally incorporate proliferation status, endocrine sensitivity, PI3K pathway alterations, and the spatial distribution of hypoxic regions (81–83).

### HER2-positive breast cancer

HER2-positive breast cancer is characterized by amplification or overexpression of the HER2 receptor and substantial activation of downstream signaling networks, particularly PI3K/AKT/mTOR and MAPK pathways. These pathways intersect with HIF signaling and can reinforce cellular adaptation to oxygen limitation. HER2 activation can enhance glucose uptake, glycolysis, biosynthetic metabolism, and cellular proliferation. Under hypoxic conditions, HIF signaling may further increase glycolytic activity while redirecting mitochondrial metabolism and promoting survival. This convergence between HER2 signaling and oxygen-stress responses can create a highly adaptable metabolic phenotype. Hypoxia may also influence the efficacy of HER2-directed therapies. Although HER2 inhibition can produce substantial tumor regression, oxygen-stressed subpopulations may survive through activation of downstream signaling pathways, metabolic switching, autophagy, and alternative receptor-mediated survival mechanisms. The ability to maintain PI3K/AKT/mTOR activity despite HER2 blockade is particularly relevant because this pathway can function as a bypass mechanism. Mitochondrial and redox adaptation may further contribute to resistance. HER2-driven tumors can exhibit high metabolic activity and increased ROS production, creating a requirement for efficient antioxidant defenses. Hypoxic adaptation may strengthen glutathione-, thioredoxin-, and NRF2-associated pathways, allowing tumor cells to tolerate both metabolic stress and treatment-induced oxidative injury. These observations suggest that HER2-positive tumors may benefit from therapeutic approaches that simultaneously disrupt HER2 signaling and oxygen-stress adaptation. Potential strategies include combinations of HER2-directed therapy with selected inhibitors of PI3K/AKT/mTOR signaling, HIF-associated pathways, metabolic dependencies, autophagy, or redox regulation. Patient selection will be critical because not all HER2-positive tumors exhibit equivalent metabolic or hypoxic dependencies (84–86).

### Triple-negative breast cancer

Triple-negative breast cancer represents perhaps the most compelling context for studying oxygen-stress adaptation because of its aggressive biology, high cellular plasticity, substantial molecular heterogeneity, and limited dependence on a single targetable receptor pathway. TNBC frequently demonstrates pronounced metabolic remodeling, EMT, stemness, angiogenesis, immune dysregulation, and therapeutic resistance, all of which can be reinforced by hypoxia. HIF signaling in TNBC can promote glycolysis, angiogenesis, EMT, invasion, and survival. Hypoxia-induced transcriptional programs can increase expression of glucose transporters and glycolytic enzymes while facilitating lactate production. At the same time, TNBC cells can demonstrate metabolic flexibility, allowing them to switch between glycolysis, glutamine metabolism, lipid utilization, and mitochondrial respiration according to environmental conditions. The interaction between hypoxia and EMT is particularly important in TNBC. HIF-dependent activation of SNAIL, SLUG, TWIST, and ZEB-associated pathways can promote epithelial–mesenchymal plasticity, increasing migration and invasion. Hypoxia may also enrich for hybrid epithelial/mesenchymal states that combine proliferative and migratory characteristics. Such phenotypic plasticity can facilitate metastatic dissemination and enable tumor cells to adapt rapidly following therapy. Hypoxia may additionally reinforce TNBC stemness. HIF signaling can interact with Notch, Wnt/β-catenin, and other developmental pathways to maintain tumor-initiating populations. These cells may possess enhanced DNA repair, antioxidant capacity, drug-efflux activity, and metabolic flexibility, allowing them to survive chemotherapy and subsequently regenerate tumor heterogeneity. The immune consequences of hypoxia are also highly relevant to TNBC because a substantial subset of these tumors has an active immune microenvironment and may be considered for immunotherapy. Hypoxia can impair cytotoxic T-cell and natural killer-cell function, increase immunosuppressive signaling, promote macrophage reprogramming, and increase extracellular adenosine. Consequently, a tumor may exhibit substantial immune infiltration while remaining functionally resistant to immune-mediated killing (87–89).

### Basal-like and other molecularly defined subgroups

The conventional clinical classification of breast cancer into HR-positive, HER2-positive, and TNBC categories does not completely capture oxygen-stress heterogeneity. Gene-expression profiling identifies additional biological groups, including basal-like, luminal, HER2-enriched, and other molecular states. Basal-like tumors often overlap substantially with TNBC but represent a molecular rather than purely receptor-defined category. Their biology is frequently characterized by high proliferation, genomic instability, metabolic stress, and pronounced cellular plasticity. These characteristics may increase dependence on adaptive pathways that control DNA repair, ROS, mitochondrial function, and proteostasis during oxygen stress. Within HER2-enriched tumors, the magnitude of HER2 signaling, PI3K pathway activity, metabolic phenotype, and immune contexture can determine the response to hypoxia. Similarly, luminal tumors can differ markedly in proliferation, endocrine dependence, metabolic activity, and hypoxia-associated signaling (90).

### Subtype-specific differences in metabolic adaptation

An important distinction among breast cancer subtypes is how oxygen stress modifies energy metabolism. Hypoxic cells generally increase glycolysis, but the magnitude of this response and the availability of compensatory pathways vary considerably. TNBC may demonstrate particularly strong dependence on glycolytic and alternative nutrient pathways, whereas some HR-positive tumors retain greater oxidative metabolic capacity. HER2-positive tumors can combine receptor-driven anabolic signaling with substantial metabolic remodeling. This heterogeneity creates both opportunities and challenges. A glycolysis inhibitor may be effective in a glycolysis-dependent tumor but ineffective in a metabolically flexible tumor capable of switching to oxidative phosphorylation or fatty acid metabolism (91).

### Subtype-specific differences in redox adaptation

All breast cancer subtypes must control ROS during hypoxia and reoxygenation, but their antioxidant dependencies can differ. Hypoxic tumor cells can increase glutathione and thioredoxin activity, NRF2 signaling, and NADPH production. TNBC, particularly aggressive tumors with substantial metabolic activity, may experience high baseline oxidative stress and therefore develop strong antioxidant defenses. HER2-driven signaling may similarly increase metabolic ROS production and select for efficient redox buffering. These features create a potential therapeutic vulnerability. Tumors with high oxidative stress and strong dependence on glutathione or thioredoxin systems may be particularly susceptible to treatments that increase ROS while simultaneously disabling antioxidant compensation (91–93).

### Therapeutic implications in breast cancer

The presence of hypoxia within the breast tumor microenvironment presents a major challenge to conventional cancer therapies, but it also offers opportunities for novel, targeted interventions (59). Hypoxia-induced adaptations—such as metabolic reprogramming, angiogenesis, and resistance to apoptosis—not only enable tumor survival and progression but also confer resistance to chemotherapy, radiotherapy, and hormonal treatments (59, 60). One therapeutic avenue involves the inhibition of hypoxia-inducible factors (HIFs), particularly HIF-1α, which acts as a master regulator of the cellular hypoxic response. Preclinical studies have demonstrated that HIF-1α inhibition can reduce tumor angiogenesis, sensitize breast cancer cells to radiation, and suppress metastasis. Small-molecule inhibitors of HIF-1α, such as PX-478, are currently under investigation and have shown promising anti-tumor activity in early-phase clinical trials. However, due to the complexity and redundancy of hypoxia signaling pathways, targeting HIFs alone may not be sufficient in all tumor contexts (61, 62). Another promising strategy targets the altered metabolism of hypoxic breast cancer cells. Glycolytic inhibitors, such as 2-deoxy-D-glucose (2-DG), and lactate transport inhibitors targeting monocarboxylate transporters (MCTs) are being explored to disrupt energy production in hypoxic cells. Similarly, drugs that modulate mitochondrial function or block glutamine metabolism may selectively impact hypoxia-adapted cancer cells. Importantly, these agents may enhance the efficacy of standard therapies by sensitizing hypoxic cells that are otherwise resistant (63, 64).

Angiogenesis inhibitors, such as the anti-VEGF monoclonal antibody bevacizumab, have been tested in breast cancer with mixed results. While they can transiently normalize tumor vasculature and improve drug delivery, long-term use may paradoxically exacerbate hypoxia by excessively pruning blood vessels. This has led to the concept of “vascular normalization” rather than complete inhibition—using lower or intermittent doses to improve perfusion and reduce hypoxic stress. Combining angiogenesis inhibitors with chemotherapy, immunotherapy, or metabolic modulators may offer a more balanced and effective therapeutic approach (65, 66). Efforts to re-sensitize hypoxic tumor cells to apoptosis have also gained traction. BH3 mimetics, which inhibit anti-apoptotic BCL-2 family proteins, are under investigation in combination with other agents to overcome apoptosis resistance in hypoxic breast tumors. Moreover, autophagy inhibitors such as chloroquine have shown potential in disrupting hypoxia-induced survival pathways, particularly when used in combination with chemotherapy or radiotherapy (67, 68). Recent advances in immunotherapy also hold promise for overcoming hypoxia-associated immune evasion. Hypoxia can suppress immune surveillance by impairing cytotoxic T cell activity and promoting an immunosuppressive milieu. Strategies to reverse these effects include the use of immune checkpoint inhibitors alongside agents that normalize the tumor vasculature or modulate the tumor’s metabolic landscape. Targeting the hypoxic niche may thus enhance the efficacy of immunotherapeutic regimens in breast cancer, particularly in more aggressive or treatment-refractory subtypes (Figure 3) (69, 70).

**FIGURE 3:**
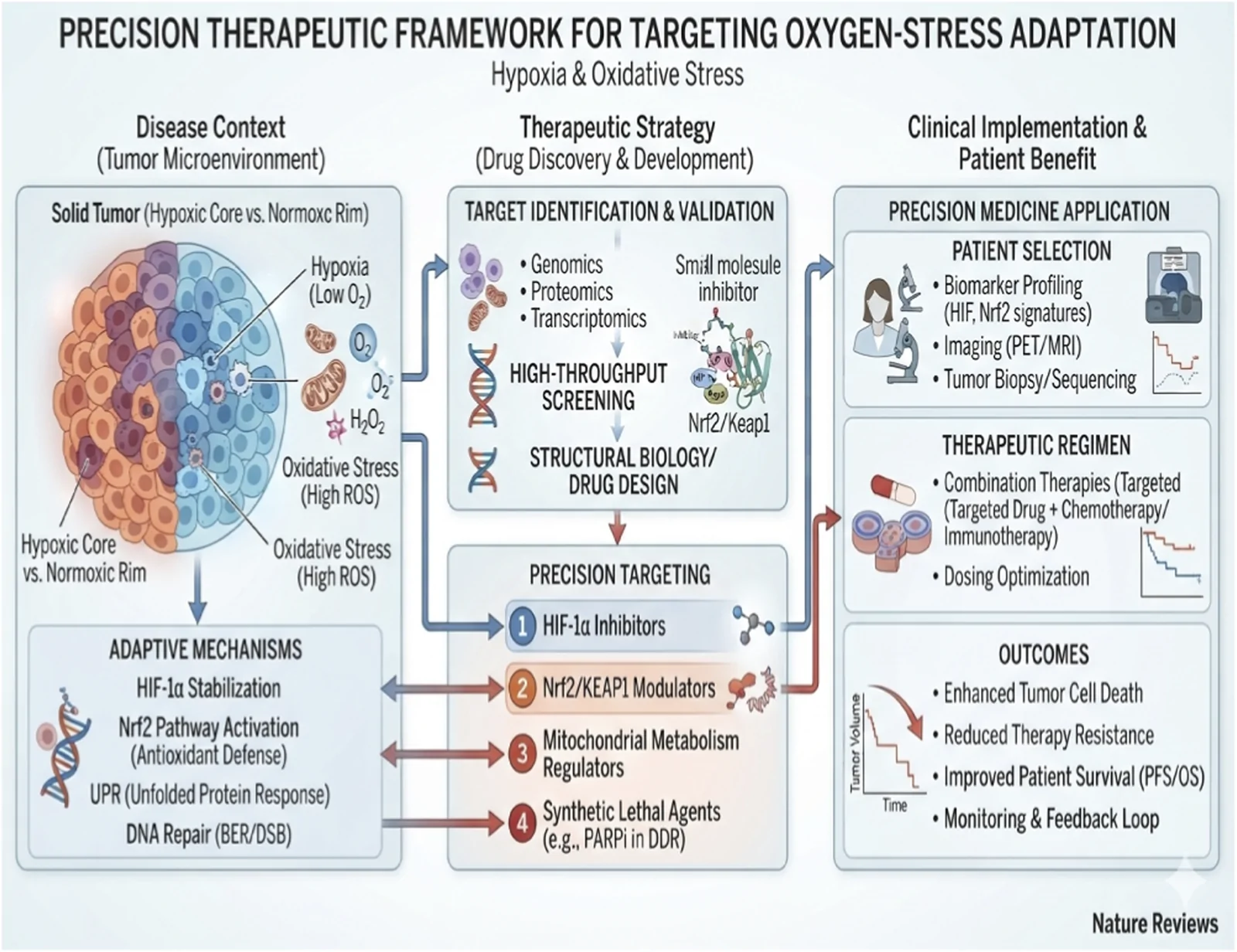
Precision therapeutic framework for targeting oxygen-stress adaptation.

## Conclusion

Oxygen stress, particularly tumor hypoxia, is a central and dynamic determinant of breast cancer biology. Rather than representing a passive metabolic constraint, hypoxia functions as a potent selective force that drives molecular reprogramming, enabling malignant cells to survive, adapt, and evolve within hostile microenvironments. Through coordinated activation of hypoxia-inducible signaling pathways, breast cancer cells enhance angiogenesis, rewire metabolism toward glycolysis, suppress immune surveillance, and acquire invasive and stem-like phenotypes that collectively promote tumor progression and metastasis. These hypoxia-driven adaptations also underpin resistance to major treatment modalities, including chemotherapy, radiotherapy, endocrine therapy, and targeted agents. This resistance arises from multiple intersecting mechanisms, such as altered drug delivery, enhanced DNA repair capacity, inhibition of apoptosis, and metabolic flexibility. As a result, oxygen stress is closely linked to poor prognosis and reduced survival outcomes in breast cancer patients.
